# Hepatocellular Carcinoma with Inferior Vena Cava and Right Atrium Tumor Thrombus

**DOI:** 10.15388/Amed.2021.28.2.10

**Published:** 2021-08-18

**Authors:** Arun Kumar Gunasekaran, Amit Malviya, Tony Ete, Animesh Mishra, Bhupen Barman, Md Jamil, Donboklang Lynser

**Affiliations:** Department of Cardiology, North Eastern Indira Gandhi Regional Institute of Health and Medical Sciences, Mawdiangdiang, Shillong, Meghalaya, India Orcid: https://orcid.org/0000-0002-7461-3518; Department of Cardiology, North Eastern Indira Gandhi Regional Institute of Health and Medical Sciences, Mawdiangdiang, Shillong, Meghalaya, India. Orcid: https://orcid.org/0000-0002-3326-6177; Department of Cardiology, North Eastern Indira Gandhi Regional Institute of Health and Medical Sciences, Mawdiangdiang, Shillong, Meghalaya, India Orcid: https://orcid.org/0000-0002-2609-4733; Department of Cardiology, North Eastern Indira Gandhi Regional Institute of Health and Medical Sciences, Mawdiangdiang, Shillong, Meghalaya, India. Orcid: https://orcid.org/0000-0002-8482-6613; Department of Medicine, North Eastern Indira Gandhi Regional Institute of Health and Medical Sciences, Mawdiangdiang, Shillong, Meghalaya, India Orcid: https://orcid.org/0000-0002-5433-1310; Department of Medicine, North Eastern Indira Gandhi Regional Institute of Health and Medical Sciences, Mawdiangdiang, Shillong, Meghalaya, India Orcid: https://orcid.org/0000-0003-4758-8721; Department of Radiology and Imaging, North Eastern Indira Gandhi Regional Institute of Health and Medical Sciences, Mawdiangdiang, Shillong, Meghalaya, India Orcid: https://orcid.org/0000-0001-5525-2422

**Keywords:** Carcinoma, hepatocellular, Thrombosis, Echocardiography

## Abstract

Hepatocellular carcinoma (HCC) is one of the leading causes of cancer and cancer related deaths worldwide. Metastasis of HCC into the cardiac cavity is mostly caused by direct tumor thrombus invasion through the major hepatic veins and of vena cava inferior with continuous extension into the right cardiac cavity. Right heart metastasis without invasion of inferior vena cava (IVC), which may be caused by haematogenous spread of cancer cells, is rarely reported. We report a case of HCC with IVC and right atrium (RA) thrombus in a patient who presented to us with decompensated cardiac failure. Strikingly, the patient was young and with negative serum HBsAg, and anti-HCV results. Our case highlights a rare presentation of metastatic intracardiac tumor thrombus involving the RA in advanced HCC without any symptoms of cardiac failure, and henceforth, the role of screening echocardiography for all patients with advanced HCC especially with vena caval involvement to rule out intracardiac thrombus.

## Abstract (lt)

**Hepatoceliulinė karcinoma ir apatinės tuščiosios venos bei dešiniojo prieširdžio navikinis trombas:**

**Santrauka. **Hepatoceliulinė karcinoma (HCK) yra viena iš dažniausių vėžio ir su vėžiu susijusių mirčių priežastis visame pasaulyje. Širdies ertmės HCK metastazes dažniausiai sukelia navikinis trombas, įsiskverbęs per kepenų venas ir apatinę tuščiąją veną ir toliau judantis link dešiniosios širdies ertmės. Metastazių dešinėje širdies ertmėje, kai nėra įsiskverbta į apatinę tuščiąją veną (ATV) – tokį įsiskverbimą gali sukelti vėžinių ląstelių plitimas, – pasitaiko retai. Pranešame apie HCK ir ATV bei dešiniojo prieširdžio (DP) trombo atvejį, kurį nustatėme kartu su dekompensuotu širdies nepakankamumu. Mūsų nuostabai pacientas buvo jaunas, o jo hepatito B viruso paviršinio antigeno (HbsAg) ir hepatito C tyrimo rezultatai buvo neigiami. Aprašome retą metastazinį širdies navikinio trombo DP atvejį, sergant vėlesnės stadijos HCK ir nepasireiškus jokiems širdies nepakankamumo simptomams, bei pabrėžiame pacientų, sergančių vėlesnės stadijos HCK echokardiografijos tyrimo svarbą, ypač kai pažeista tuščioji vena, siekiant atmesti širdies trombo galimybę.

**Raktažodžiai:** karcinoma, hepatoceliulinė karcinoma, trombozė, echokardiografija

## Introduction

Hepatocellular carcinoma (HCC) is the sixth leading cause of cancer in the world and the second leading cause of cancer-related deaths in the world [1]. Common causes for HCC are chronic viral hepatitis due to hepatitis B virus or hepatitis C virus infection and chronic alcohol consumption. Chronic alcohol use of more than 80 g/day for more than 10 years increases the risk for HCC by approximately 5-fold. The postulated mechanisms by which alcohol causes HCC are not known, but have been hypothesized to include direct cancer-promoting effects of alcohol and acetaldehyde, genetic susceptibility, oxidative stress and immune dysregulation by changes in retinoic acid metabolism and DNA methylation [2]. Other common risk factors for the development of HCC are obesity related liver disease (nonalcoholic steatohepatitis), autoimmune hepatitis, inherited metabolic diseases like Wilson’s disease, hemochromatosis or alpha1-antitrypsin deficiency, and mycotoxin contamination of food stuffs (aflatoxin B1). HCC is an aggressive tumor and can show extensive metastasis [3]. HCC often causes portal vein tumour thrombus formation, with metastasis to the liver, lungs, and other organs, while the direct invasion of the hepatic vein and formation of a tumor thrombus extending into the IVC and RA is relatively rare. As a cause of secondary Budd–Chiari syndrome, HCC has been even more rarely reported [4,5].

In this report, we present the rare case of an advanced HCC with invasion of the inferior vena cava (IVC), portal vein, and intravascular extension to the right atrium (RA) in a patient with preexisting liver disease.

## Case report

A 32-year-old man, a known case of alcoholic lever disease, presented with chief complaints of abdominal pain and distension for 6 months and bilateral leg swelling for past one month. History of loss of weight and loss of appetite were present. On examination in the emergency department, the temperature was 36.7°C, the blood pressure 122/81 mm Hg, the pulse 76 beats per minute, the respiratory rate 18 breaths per minute, and the oxygen saturation 92% while the patient was breathing ambient air. He was alert and oriented and had no asterixis. The skin was jaundiced, the sclerae were icteric. The skin was without spider angiomas or palmar erythema. Bilateral pedal oedema was present. The first and second heart sounds were normal and grade 3/6 systolic murmur was present in lower left sternal border, and the jugular venous pulsation was distended. The abdomen was distended and fluid thrill was present. 

**Figure 1.**
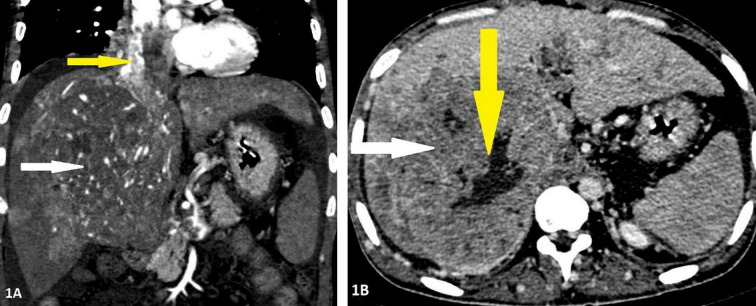
Contrast enhanced computed tomography of 32-years-old male with Hepatocellular carcinoma in the right lobe. Panel 1A: Arterial phase imaging in coronal reconstruction showing the mass having internal areas of arterial hyperenhancement (white arrow) with enhancing tumoral thrombus in the inferior vena cava and right atrium (yellow arrow). Panel 1B: Portal phase imaging in axial section showing washout of contrast in the lesion (white arrow) with central area of nonenhancement suggestive of necrosis (yellow arrow).

**Figure 2.**
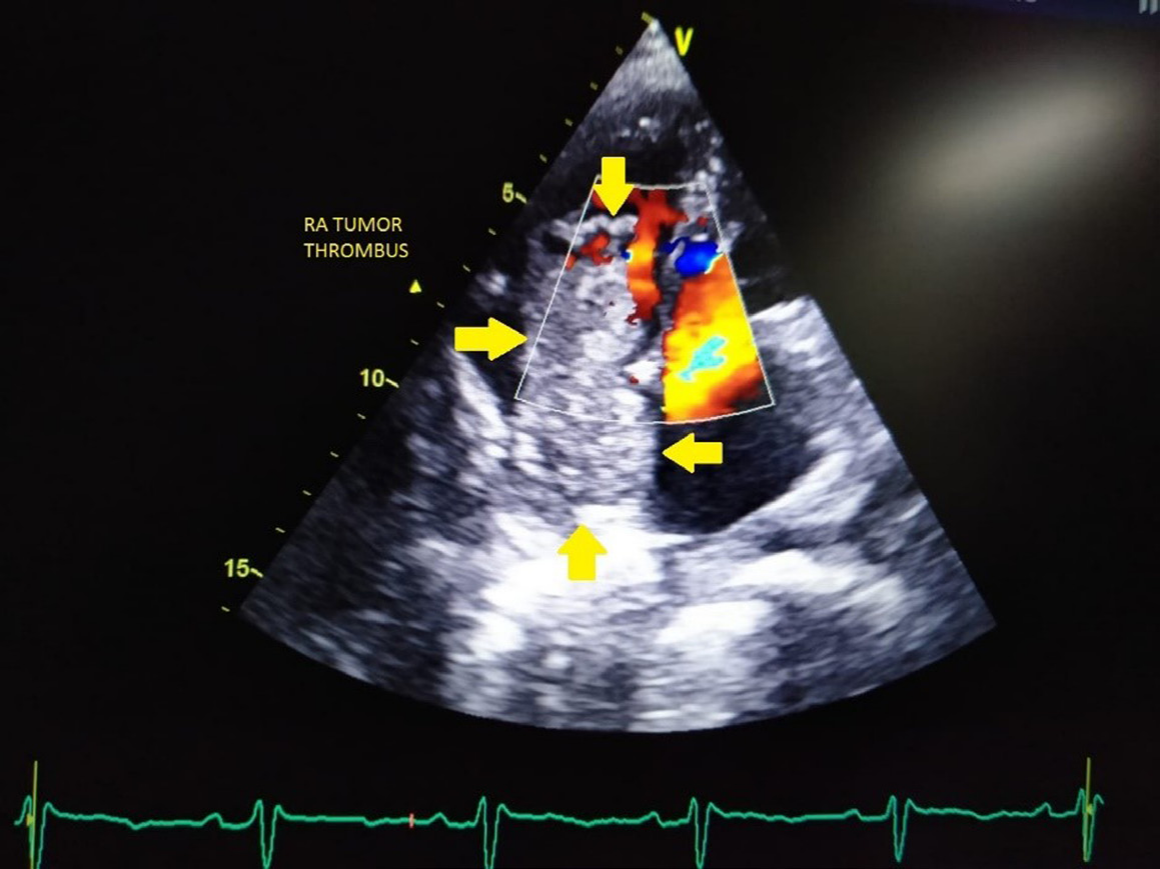
Transthoracic echocardiography image showing a large intracardiac right atrial thrombus measuring 5.7 × 2.4 cm obstructing the tricuspid valve during the diastolic phase of the cardiac cycle (RA; arrow).

His blood tests for hepatitis B virus (HBV) surface antigen, hepatitis B virus (HBV) core IgM antibodies, hepatitis C virus (HCV) antibodies, and human immunodeficiency virus (HIV) type 1 (HIV-1) p24 antigen and HIV-1 and HIV-2 antibodies were negative. Other laboratory results (normal ranges in parentheses) were as follows: Haemoglobin, 9.4 gm/dl (12–15 gm/dl); total bilirubin, 12 mg/dl (0.2–1.2 mg/dl); aspartate aminotransferase, 376 IU/l (5–40 IU/l); alanine aminotransferase, 114 IU/l (5–47 IU/l); alkaline phosphatase, 444 IU/l (111–295 IU/l); and lactate dehydrogenase, 229 IU/l (100–225 IU/l). Ultrasonography of the abdomen revealed a large space-occupying lesion in the right lobe of the liver with the background of chronic parenchymal liver disease and portal vein thrombosis. Contrast Enhanced Computed Tomography (CECT) abdomen reveals an 11.9 × 10.8 × 14 cm hypervascular mass showing enhancement on arterial phase and rapid washout in the right lobe of liver with thrombus extending into IVC and portal vein (Figure 1). The serum α-fetoprotein (AFP) was elevated to > 54000 ng/ml (normal range <13.2 ng/ml). His electrocardiography showed normal sinus rhythm and chest X-ray showed mild right-sided pleural effusion and normal cardiothoracic ratio. A 2D transthoracic echocardiogram demonstrated a large intracardiac right atrial thrombus measuring 5.7 × 2.4 cm obstructing the tricuspid valve during the diastolic phase of the cardiac cycle (Figure 2).

The patient was reevaluated for further management course after reaching the final diagnosis of HCC. His condition was reviewed by medicine, oncology, cardiology and cardiothoracic team and he was started on chemotherapy (Sorafenib 400mg daily), heparin bridge therapy with warfarin with a plan to resect the mass extending to the right atrium. But the patient’s relatives were opted for conservative management only and were discharged after initial stabilization. Patient came for follow up after one month and he was doing well. 

## Discussion

HCC usually metastasizes to regional lymph nodes, lungs or bones, and also has a high propensity to involve local vascular and endovascular involvement [4]. According to the literature and autopsy studies, invasion of the IVC has been described in up to 9–26% and intravascular extension to the RA in 1–4% of cases of HCC [5-6]. However the incidence of vascular invasion is increased about 82% when the tumor size is more than 5 cm and serum AFP levels were more than 1000 ng/ml [7]. The mechanism of cardiac metastases is as a contiguous extension from the intrahepatic HCC via a tumor thrombus to the IVC or by lymphatic or hematogenous spread. The majority of cardiac metastases are direct and contiguous extensions of the intrahepatic HCC.

The RA tumor thrombus may cause sudden hemodynamic collapse by tricuspid valve obstruction, secondary Budd–Chiari syndrome, right heart failure, and massive pulmonary thromboembolism [8]. The patients with HCC complicating RA tumor thrombus have a poor prognosis. If not treated, the survival time is between three days and two months [9]. The early diagnosis and treatment can prolong the survival of patients. Currently, simultaneous surgical removal of RA thrombus and resection of HCC may prolong the survival of more than 2 years [10]. In a retrospective cohort study, Wang et al. concluded that in the treatment of HCC extending into the IVC/RA, hepatectomy and thrombectomy group had a median survival of 19 months, the TACE group had a median survival of 4.5 months, and symptomatic treatment had a median survival of 5 months. These data indicated that surgery, either removing thrombus combined with hepatectomy or only tumor thrombus extraction, might result in better survival when compared with other nonsurgical therapies [11]. Our patient refused surgery and the long term follow up is needed. Since very few patients are responsive to local surgery and nonsurgical therapies like TACE, local radiotherapy is also moderately effective for HCC. The cancer chemotherapeutics are very little effective in advanced HCC, so the immunomodulators like sorafenib are used very often. These immunomodulators are shown to prolong survival benefit and slow down the radiologic progression of HCC [12].

## Conclusion

HCC with tumor thrombus extending into the right atrium is frequently associated with secondary Budd–Chiari syndrome and often other adverse tumor-related factors, such as extension to the portal veins; thus, it is traditionally regarded as a terminal condition with extremely poor prognosis. Almost all patients with HCC which develops from cirrhotic liver should be screened for IVC extension and RA tumor thrombus. The early screening and appropriate treatment modality may improve the survival of the patients. The echocardiography is a simple, noninvasive diagnostic tool for RA tumor thrombus, hemodynamics, cardiac function and pulmonary pressures. 

## References

[ref1] Desai A, Sandhu S, Lai JP, Sandhu DS. Hepatocellular carcinoma in non-cirrhotic liver: A comprehensive review. World J Hepatol. 2019 Jan 27;11(1):1–18. doi: 10.4254/wjh.v11.i1.1 30705715 PMC6354117

[ref2] Morgan TR, Mandayam S, Jamal MM. Alcohol and hepatocellular carcinoma. Gastroenterology. 2004 Nov;127(5 Suppl 1):S87–96. doi: 10.1053/j.gastro.2004.09.020 15508108

[ref3] Caldwell S, Park SH. The epidemiology of hepatocellular cancer: from the perspectives of public health problem to tumor biology. J Gastroenterol. 2009;44 Suppl 19:96–101. doi: 10.1007/s00535-008-2258-6 19148801

[ref4] Sung AD, Cheng S, Moslehi J, Scully EP, Prior JM, Loscalzo J. Hepatocellular carcinoma with intracavitary cardiac involvement: a case report and review of the literature. Am J Cardiol. 2008 Sep 1;102(5):643–5. doi: 10.1016/j.amjcard.2008.04.042 18721529

[ref5] EDMONDSON HA, STEINER PE. Primary carcinoma of the liver: a study of 100 cases among 48,900 necropsies. Cancer. 1954 May;7(3):462–503. doi: 10.1002/1097-0142(195405)7:3<462::AID-CNCR2820070308>3.0.CO;2-E 13160935

[ref6] Kato Y, Tanaka N, Kobayashi K, Ikeda T, Hattori N, Nonomura A. Growth of hepatocellular carcinoma into the right atrium. Report of five cases. Ann Intern Med. 1983 Oct;99(4):472–4. doi: 10.7326/0003-4819-99-4-472 6312861

[ref7] Sakata J, Shirai Y, Wakai T, Kaneko K, Nagahashi M, Hatakeyama K. Preoperative predictors of vascular invasion in hepatocellular carcinoma. Eur J Surg Oncol. 2008 Aug;34(8):900–5. doi: 10.1016/j.ejso.2008.01.031 18343084

[ref8] Chan GS, Ng WK, Ng IO, Dickens P. Sudden death from massive pulmonary tumor embolism due to hepatocellular carcinoma. Forensic Sci Int. 2000 Feb 28;108(3):215–21. doi: 10.1016/s0379-0738(99)00212-1 10737468

[ref9] Pesi B, Giudici F, Moraldi L, Montesi G, Romagnoli S, Pinelli F, et al. Hepatocellular carcinoma on cirrhosis complicated with tumoral thrombi extended to the right atrium: results in three cases treated with major hepatectomy and thrombectomy under hypothermic cardiocirculatory arrest and literature review. World J Surg Oncol. 2016 Mar 12;14:83. doi: 10.1186/s12957-016-0831-7 PMC4789284 26971195

[ref10] Inoue Y, Hayashi M, Katsumata T, Shibayama Y, Tanigawa N. Hepatocellular carcinoma with right atrial tumor thrombus: report of a case. Surg Today. 2011 Aug;41(8):1122–9. 10.1007/s00595-010-4443-5 21773904

[ref11] Wang Y, Yuan L, Ge RL, Sun Y, Wei G. Survival benefit of surgical treatment for hepatocellular carcinoma with inferior vena cava/right atrium tumor thrombus: results of a retrospective cohort study. Ann Surg Oncol. 2013 Mar;20(3):914–22. doi: 10.1245/s10434-012-2646-2 22956071

[ref12] Llovet JM, Ricci S, Mazzaferro V, Hilgard P, Gane E, Blanc JF, et al; SHARP Investigators Study Group. Sorafenib in advanced hepatocellular carcinoma. N Engl J Med. 2008 Jul 24;359(4):378–90. 10.1056/NEJMoa0708857 18650514

